# Fatty Acid Binding Protein 11a Is Required for Brain Vessel Integrity in Zebrafish

**DOI:** 10.3389/fphys.2017.00214

**Published:** 2017-04-11

**Authors:** Jie Zhang, Jialing Qi, Shuilong Wu, Lijiao Peng, Yunwei Shi, Jinxian Yang, Zhenhua Yin, Yu Gao, Chengniu Wang, Jie Gong, Haijun Zhang, Jingjing Zhang, Dong Liu

**Affiliations:** ^1^Medical School of Nantong UniversityNantong, China; ^2^Affiliated Hospital of Guangdong Medical UniversityZhanjiang, China; ^3^Key Laboratory of Neuroregeneration of Jiangsu and Ministry of Education, Co-innovation Center of Neuroregeneration, Nantong UniversityNantong, China; ^4^Xinglin College of Nantong UniversityNantong, China; ^5^School of Life Science, Nantong UniversityNantong, China; ^6^Laboratory Animal Center, Nantong UniversityNantong, China

**Keywords:** FABP4, *fabp11a*, vessel permeability, knockout, zebrafish

## Abstract

The monolayer of endothelial cells (ECs) lining the intima of all blood vessel wall forms a semipermeable barrier that regulates tissue-fluid homeostasis, transport of nutrients, and migration of blood cells across the barrier. A number of signaling pathways and molecules mediate endothelial permeability, which plays important roles in a variety of the physiological and pathological conditions. Fatty acid binding proteins (FABPs) are able to bind various hydrophobic molecules, such as long-chain fatty acids, prostaglandins and eicosanoids. FABP4, a member of the family of FABPs, plays an important role in maintenance of glucose and lipid homeostasis as well as angiogenesis. In the present study, we found that *fabp11a*, the ortholog of mammalian FABP4, was highly expressed in developing brain vessels of zebrafish. Knockout of *fabp11a* gene caused hemorrhage in zebrafish brain. Morpholino mediated *fabp11a* gene knockdown phenocopied the hemorrhage in mutants. Furthermore, we demonstrated permeability of brain vessels in *fabp11a* mutant is significantly higher than that of control. In addition, COX and LOX inhibition partially rescued the brain vessel integrity defects caused by *fabp11a* loss-of-function, suggesting the integrity defect was relevant to the Fatty Acid function.

## Introduction

The vascular system of vertebrates composed of a well-organized and hierarchical network of blood vessels, including arteries, veins, and capillaries. This network overspreads throughout every tissue and organ of the body and is adapt to the physiological function in local microenvironment (Risau, 1997; Carmeliet and Tessier-Lavigne, 2005; Larrivee et al., 2009). The development of vascular system undergoes two sequential processes, vasculogenesis and angiogenesis (Risau, 1997; Carmeliet and Tessier-Lavigne, 2005; Larrivee et al., 2009). Vasculogenesis is a *de novo* formation of the blood vessels, whereas angiogenesis is the outgrowth of endothelial cells (ECs) to form new branches from a pre-existing vasculature (Risau and Flamme, 1995; Risau, 1997). The monolayer of endothelium lining the intima of all blood vessel wall forms a semipermeable barrier that regulates tissue-fluid homeostasis, transport of nutrients, and migration of blood cells across the barrier. A number of signaling pathways and molecules mediate endothelial permeability, which is important in a variety of the physiological and pathological conditions (Komarova et al., 2017).

Fatty acid binding protein 4 (FABP4, adipocyte-FABP, aP2), plays an important role in maintenance of glucose and lipid homeostasis as well as inflammation through its actions in adipocytes and macrophages (Kuwano et al., 1993; Li and Wilson, 1997), belongs to a family of intracellular FABPs. There are 9 highly conserved FABP family members which are cytosolic proteins with small molecular weight around 15 kDa. FABP members are expressed specifically in different tissues with some overlaps (Hertzel and Bernlohr, 2000). FABPs function in binding to a variety of hydrophobic ligands, such as long-chain fatty acids, prostaglandins, leukotrienes, and eicosanoids (Haunerland and Spener, 2004; Makowski and Hotamisligil, 2005). Compared with other pan-endothelial cell markers such as CD31, the expression of FABP4 is restricted to microvascular and small vascular ECs, which contributes to angiogenic responses, such as cell proliferation and migration (Elmasri et al., 2012). Recent studies in mice have shown that endothelial FABP4 are involved in some angiogenesis-related processes, such as EC survival, migration, and angiogenic sprouting (Elmasri et al., 2012). However, the developmental role of FABP4 in vasculature remains largely unknown.

The zebrafish (*Danio rerio*) has been shown by large amounts of studies as a powerful *in vivo* vertebrate model system for the study of vasculogenesis and angiogenesis (Weinstein, 2002). Combined with the transgenic methods to label the vascular ECs with fluorescent proteins, it allows high-resolution imaging of blood vessel in live embryos (Lawson and Weinstein, 2002; Wang et al., 2016). Recently, arising reverse genetic tools, such the antisense morpholino oligonucleotide (MO)-based gene knockdown strategies (Nasevicius and Ekker, 2000; Eisen and Smith, 2008) and genomic editing techniques (Huang et al., 2011; Zhu et al., 2011; Bedell et al., 2012; Gupta et al., 2012; Chang et al., 2013; Hwang et al., 2013), have been widely applied in manipulating the gene expression and function in zebrafish (Beis and Stainier, 2006). In this study, we examined the role of Fabp4 in blood vessel development in zebrafish. Current study provides new insight into the role of fatty acid binding protein in blood vessel.

## Materials and methods

### Zebrafish and ethics statements

All animal experimentation was carried out in accordance with the NIH Guidelines for the care and use of laboratory animals (http://oacu.od.nih.gov/regs/index.htm) and ethically approved by the Administration Committee of Experimental Animals, Jiangsu Province, China [Approval ID: SYXK (SU) 2007–0021]. Care and breeding of zebrafish was carried out essentially as we previously described (Wang et al., 2016). AB and transgenic zebrafish lines: *Tg(kdrl:EGFP)* and *Tg(gata1:DsRed)* used were described in our previous work (Wang et al., 2016).

### Bioinformatics

The zebrafish *fabp11a* exons information is got from Ensembl (http://asia.ensembl.org/Danio_rerio/Transcript/Summary?db=core;g=ENSDARG00000017299;r=19:32579389-32581848;t=ENSDART00000021798). The protein molecular weight is calculated using Protein Molecular Weight Calculator (http://www.sciencegateway.org/tools/proteinmw.htm). And the phylogenetic tree was built using PhyML software (Guindon et al., 2010). The protein sequences alignment were performed using T-coffee and edited by Jalview (Waterhouse et al., 2009; Di Tommaso et al., 2011).

### Gene expression analysis by quantitative PCR (qRT-PCR)

Total RNA of zebrafish embryos was isolated with Trizol (Invitrogen) and stored at –80°C. The total amount of extracted RNA was measured by Nanodrop. Genomic cDNA was then synthesized using the Transcriptor First Strand cDNA Synthesis Kit (Roche). The PCR amplifications were carried out in a total volume of 20 or 50 μl with specific primers (Supplementary Table 1) and Advantage 2 Polymerase Kit (Clontech). The primers for Real-time PCR analysis of fabp11a was described in our previous work (Qi et al., 2016). Quantitative PCR was carried out in triplicate using the FastStart Universal SYBR Green Master Mix (Roche Applied Science) on a real-time PCR detection system (StepOne™ Real-Time PCR Systems).

### Whole-mount *in situ* hybridization

The procedure for whole-mount *in situ* hybridization was carried out as described previously (Huang et al., 2013). The antisense RNA probe used was described in our previous work (Qi et al., 2016).

### Morpholino microinjection

Morpholino (Gene Tools) were resuspended in DNase/RNase-free distilled water at a stock concentration of 1 mM according to the manufacturer's instruction and stored at −20°C. MOs were diluted to 0.3 mM and injected into one-cell stage embryos. The sequences of the morpholino targeting *mfsd2aa* used was (named: *mfsd2aa*-MO): 5′-ACCATTTTCCCGAATAATGATGCTC-3′. The sequence of morpholino targeting *fabp11a* was listed in previous work (Qi et al., 2016).

### Drug treatment and *O*-dianisidine staining

Rapamycin, L-NAME and SB203580 LY294002, inhibitors of m-TOR, eNOS and P38 respectively, were purchased from Sigma-Aldrich. Rapamycin dissoveled in DMSO and was used at a final concentration of 10 nM. L-NAME and SB203580 were dissolved in tank water and were used at a final concentration of 10 uM. The inhibitors of Cyclooxygenase (COX) indomethoinc and Lornoxican were used at the final concentration 28 nM and 16 nM respectively. The inhibitor of Lipoxygenase (LOX) nordihyroguaria-retic acid (NDGA) was used at the final concentration 14 uM. *O*-dianisidine staining for globin was completed as described previously (Jiang et al., 2009).

### Imaging and microangiography

For confocal imaging, *Tg(kdrl:EGFP)* or *Tg(gata1:DsRed)* transgenic lines were used to investigate the blood vessel development. Embryos were anesthetized with egg water containing 0.16 mg/mL tricaine and 1% 1-phenyl-2 thiourea (Sigma), and mounted in 0.6% low melting agarose. Confocal images was taken with a Leica TCS-SP5 LSM. Analysis was performed using Imaris software. 10 kD and 40 kD dextran was injected into the heart, and fluorescence was visualized by confocal imaging. For the results of *in situ* hybridization and *Tg(gata1:DsRed)* and zebrafish embryos, images were acquired with an Olympus DP71 camera on an Olympus stereomicroscope MVX10.

### Statistics

Statistical analysis was performed using GraphPad Prism® version 6.0c. Fisher's exact test, χ^2^-test and Student's *t*-test were used (*P* < 0.05).

## Results

### *fabp11a* is highly conserved across vertebrates

**Figure 1:**
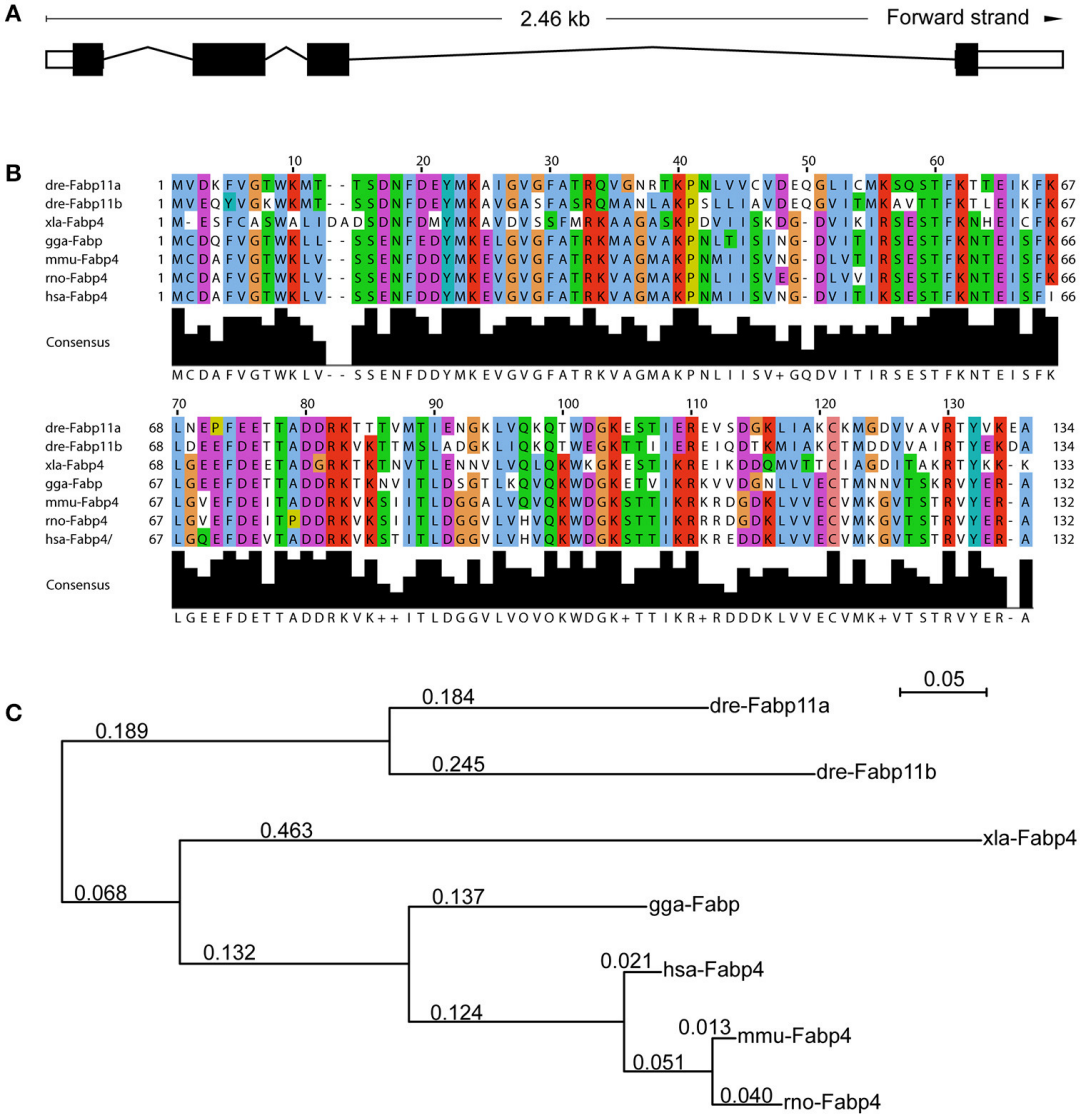
**Functional domains of Fabp11a are highly conserved during vertebrates. (A)** The *fabp11a* gene is composed of 4 exons spanning around 2.46 KB. **(B)** Alignment of Fabp11a motor domain amino acid residue sequences of *Danio rerio* (NP_001004682.1), *Danio rerio* (AAI65498.1) Fabp11b, *Gallus gallus* FABP4 (NP_989621.1), *Xenopus laevis* FABP4 (NP_001089252.1), *Mus musculus* FABP4 (CAJ18597.1), *Rattus norvegicus* FABP4 (AAH84721.1), and *Homo sapiens* FABP4 (CAG33184.1). The sequences are retrieved from NCBI Protein sequence database. **(C)** Phylogenetic tree of amino acid sequences generated using the PhyML software.

In zebrafish, *fabp11a* gene was identified as the ortholog of mammalian *FABP4* (Liu et al., 2007; Karanth et al., 2008). A number of closely related syntenic genes in the region of the zebrafish *fabp11a* locus were identified to be conserved with human *FABP4*, confirming that zebrafish *fabp4* is the orthologous gene of mammalian *FABP4* (Liu et al., 2007). The zebrafish *fabp11a* gene localizes on chromosome 19, and is composed of 4 exons spanning around 2.46 KB (Figure 1A). According to the sequence information, it is predicted to encode a 134 amino acid protein (NCBI Reference Sequence: NP_001004682.1, *gi: 52219194*), with low molecular weight of around 15.12 KDa. Multi-alignment analyses indicated that zebrafish Fabp11a and other vertebrate FABP4 proteins have orthologous relationship (Supplementary Table 2). The sequences of FABP4 from different species of vertebrates showed significant high similarities (Figure 1B). The phylogenetic analysis of selected vertebrate FABP4 proteins shows that zebrafish (*Danio rerio*) Fabp11a is mostly closed to *Mus musculus* FABP4, followed by human FABP4 (Figure 1C). These results indicate that FABP4 protein is highly conserved during the evolution of vertebrate, suggesting the functional importance of FABP4.

### *fabp11a* is expressed in developing zebrafish cerebrovessels

**Figure 2:**
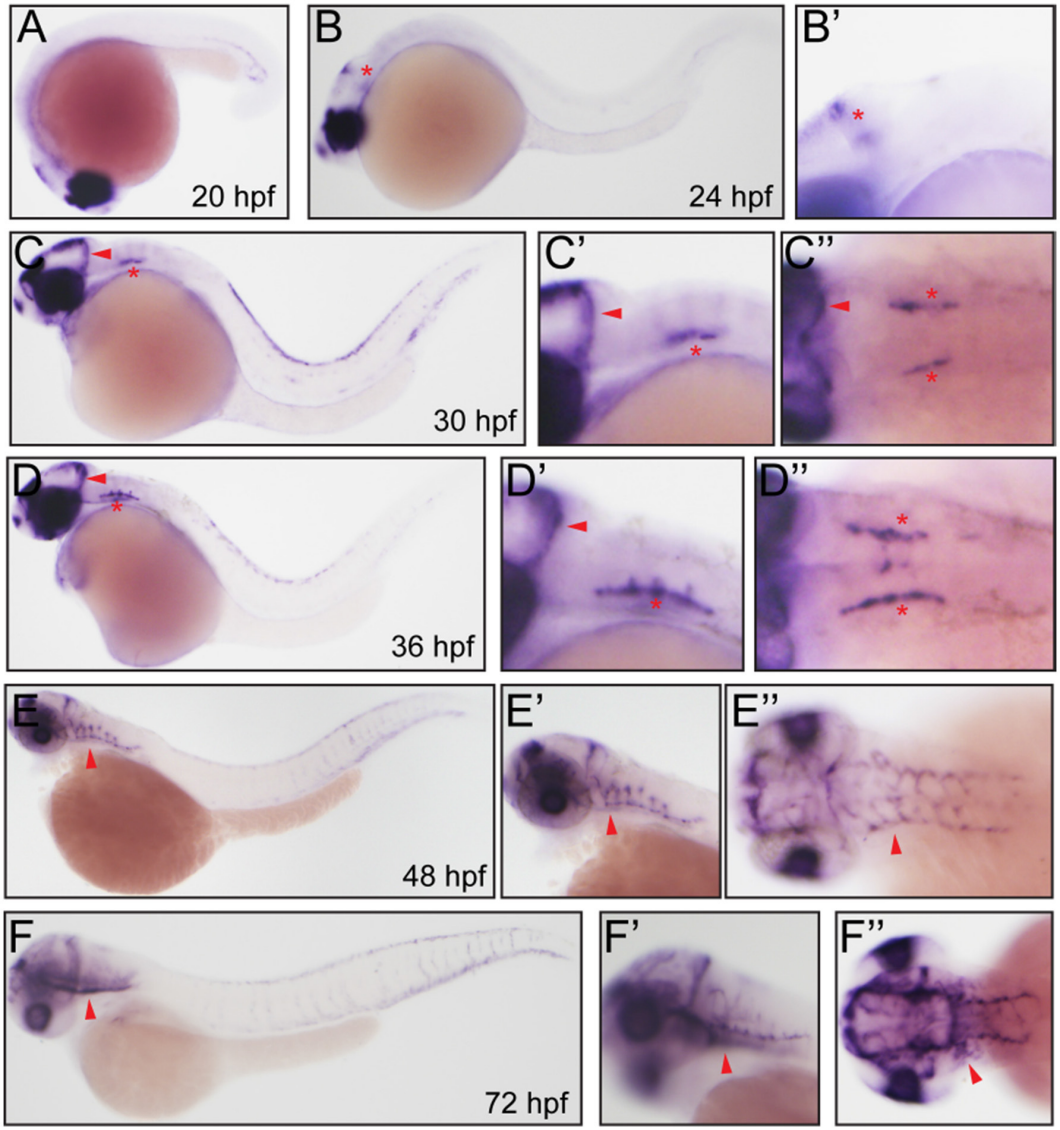
**Whole mount *in situ* hybridization analysis of *fabp11a* in zebrafish embryos. (A)** 20 hpf, lateral view. **(B)** 24 hpf, lateral view. Red asterisk indicates mid-cerebral vein (MCeV). **(B')** 24 hpf, lateral view. Red asterisk indicates mid-cerebral vein (MCeV). **(C)** 30 hpf, lateral view. Red arrowhead indicates MCeV; Red asterisk indicates primordial hindbrain channel (PHBC). **(C')** 30 hpf, lateral view. Red arrowhead indicates MCeV; Red asterisk indicates PHBC. **(C”)** 30 hpf, dorsal view. Red arrowhead indicates MCeV; Red asterisks indicate PHBC. **(D)** 36 hpf, lateral view. Red arrowhead indicates MCeV; Red asterisk indicates primordial hindbrain channel (PHBC). **(D')** 36 hpf, lateral view. Red arrowhead indicates MCeV; Red asterisk indicates PHBC. **(D”)** 36 hpf, dorsal view. Red arrowhead indicates MCeV; Red asterisks indicate PHBC. **(E,E')** 48 hpf, lateral view. Red arrowhead indicates brain vessels. **(E”)** 48 hpf, dorsal view. Red arrowhead indicates brain vessels. **(F,F')** 72 hpf, lateral view. Red arrowhead indicates brain vessels. **(F”)** 72 hpf, dorsal view. Red arrowhead indicates brain vessels.

After *in silicon* analysis of zebrafish Fabp11a, we carried out the whole-mount *in situ* hybridization experiments to analyze the temporal and spatial expression of *fabp11a* during zebrafish blood vessel development. We did not detect the expression of *fabp11a* in zebrafish blood vessels at stages earlier than 24 hpf (Figure 2A). *fabp11a* mRNA was discovered to emerge in mid-cerebral vein (MCeV) at 24 hpf (Figures 2B,B'). From 30 hpf, *fabp11a* was present in part of primordial hindbrain channel (PHBC), where central arteries (CtAs) initiate to branch, and gradually expanded to the entire PHBC and CtAs at 48 hpf (Figures 2C–E”). Additionally, we also observed that *fabp11a* was expressed in anterior cerebral Vein (ACeV) and metencephalic artery (MtA) at 72 hpf (Figures 2F–F”).

### *fabp11a* loss-of-function caused hemorrhage and impaired cerebrovascular integrity

**Figure 3:**
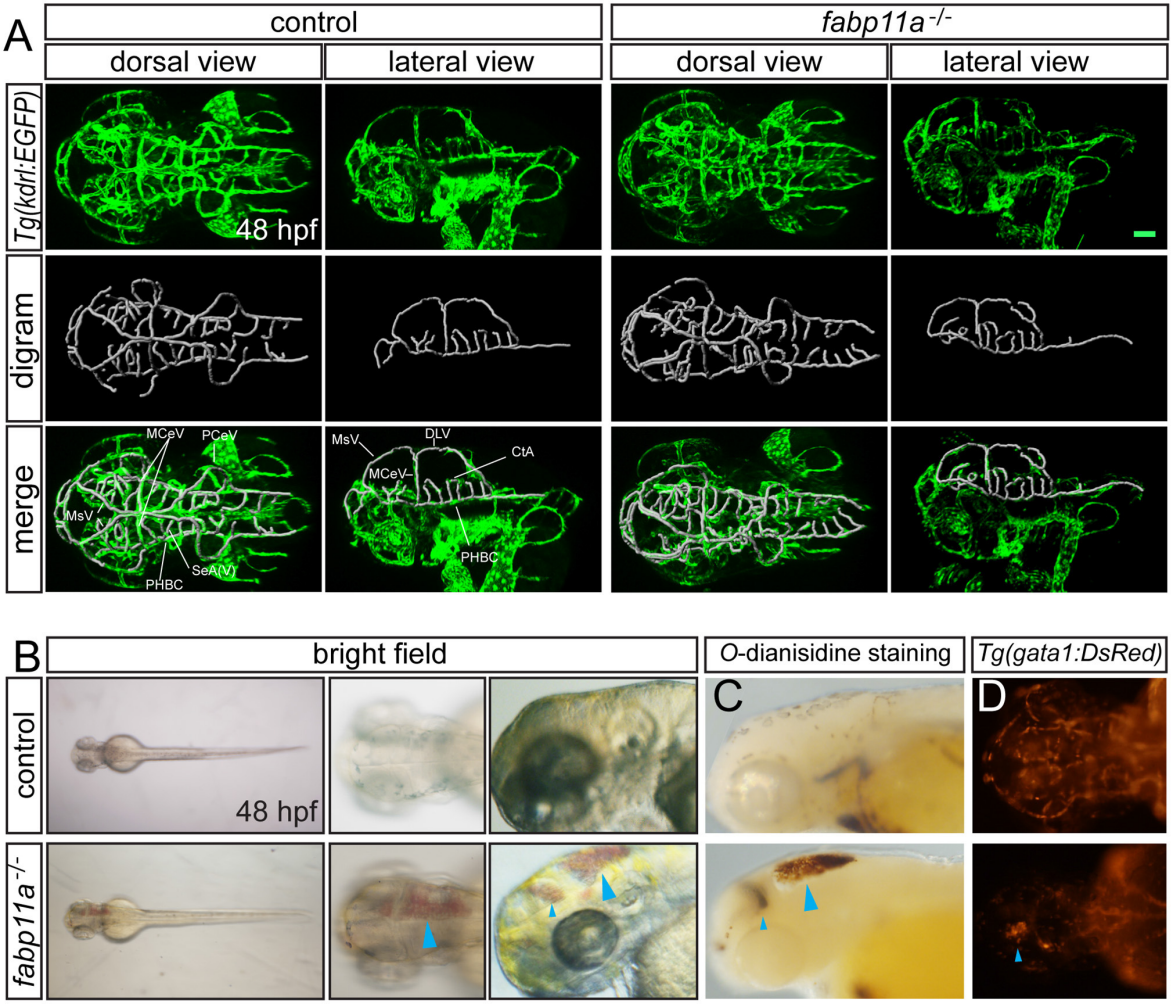
***fabp11a* knockout leads to zebrafish brain hemorrhage. (A)** Confocal imaging analysis of brain vascular morphology in control and *fabp11a* mutants *Tg*(*kdrl:EGFP*) embryos at 48 hpf. **(B)** Microscopy analysis of control embryos and *fabp11a* mutants in bright field at 48 hpf. Blue arrowheads indicate hemorrhage in zebrafish head. **(C)** Microscope analysis of control embryos and *fabp11a* mutants with *O*-dianisidine staining in bright field at 48 hpf. Blue arrowheads indicate hemorrhage in zebrafish head. **(D)** Fluorescent microscopy imaging of *Tg*(*gata1:DsRed*) embryos at 48 hpf. Scale bar: 20 μm.

To uncover the biological function of *fabp11a* in zebrafish brain vessels, we did the confocal imaging analysis of *fabp11a* mutants, which were generated using CRISPR-associated (Cas) 9 system (CRISPR/Cas9), as previously described (Qi et al., 2016), on *Tg*(*kdrl:EGFP*) line. We did not find obvious morphological defects of the brain vessels, including MCeV, PHBC, and CtAs in *fabp11a* mutants (Figure 3A). However, we found that around 22% of the *fabp11a* mutants displayed hemorrhage in the head (Figure 3B). The leaked blood cell were evidently observed in the brain after *O*-dianisidine staining (Figure 3C) or in *Tg*(*gata1:dsRed*) line (Figure 3D), in which red blood cell is labeled with red florescence. Morpholino mediated *fabp11a* gene knock down phenocopied the hemorrhage in mutants (Figures S1A–D”), which confirmed that the hemorrhage phenotype was caused by *fabp11a* loss-of-function. Furthermore, we demonstrated that the hemorrhage phenotype could be rescued by co-injection of *fabp11a* mRNA together with *fabp11a* MO (Figure S1E).

**Figure 4:**
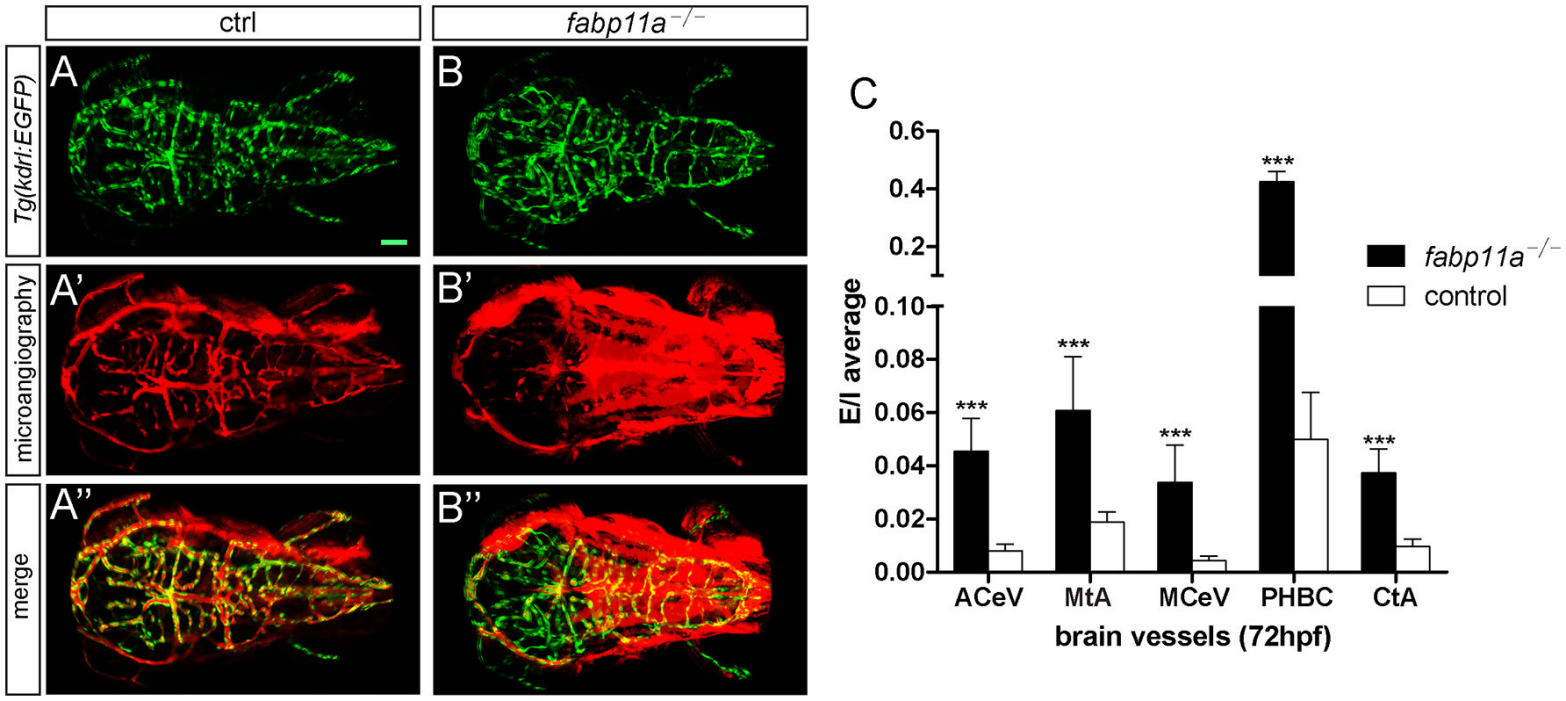
**Zebrafish *fabp11a* mutants present disrupted brain vessel integrity. (A–B”)** Confocal imaging analysis of *fabp11a* mutants and control *Tg*(*kdrl:EGFP*) embryos intracardiac injected with Rhodamine-conjugated dextran at 72 hpf. **(C)** Quantification of the permeability of the selected vessels in *fabp11a* mutants and control *Tg*(*kdrl:EGFP*) embryos using E/I = extravascular fluorescence intensity /Intravascular fluorescence intensity. *T*-test, ^***^P < 0.001, scale bar: 20 μm.

To investigate the permeability of cerebrovasculature after the loss of fabp11a, micro-injection of 10,000 and 40,000 MW Rhodamine-conjugated dextrans into the arterial vessel at 3 dpf followed by live imaging were performed. As a result, fluorescent dextran was observed being confined within vessels of control embryos (Figures 4A–A”). In contrast, dextran extravasated from cerebrovessels as evidenced by fluorescence within the brain parenchyma of fabp11a mutant larvae (Figures 4B–B”). In addition we measured the fluorescence intensity of extra- and intra-vascular and used E/I = extravascular fluorescence intensity /Intravascular fluorescence intensity to quantify the permeability of the selected vessels, where the fabp11a is expressed highly and found that permeability in fabp11a mutant is significantly higher than that of control (Figure 4C). These results suggest that fabp11a regulates brain vessel integrity in zebrafish.

### COX and LOX inhibition partially rescued the brain vessel integrity defects caused by *fabp11a* loss-of-function

A number of signaling was implicated to the role of FABP4 in angiogenesis. To understand the potential mechanisms, through which *fabp11a* regulates the brain vessel integrity, we did a serials of experiments to investigate whether these pathways got involved (Figures S2A–D). Pharmacological inhibition of mTOR signaling with rapamycin resulted in significant reduction of branching angiogenesis of brain vessels but not the hemorrhage in head (Figure S2B). We also found that L-NAME (eNOS inhibitor) and SB203580 (P38 inhibitor) treatment induced the similar phenotypes with those caused by mTOR inhibition (Figures S2C,D). Through RT-PCR analysis, we showed *fabp11a* was downregulated in the rapamycin, L-NAME, and SB203580 treated embryos (Figure S2E).

**Figure 5:**
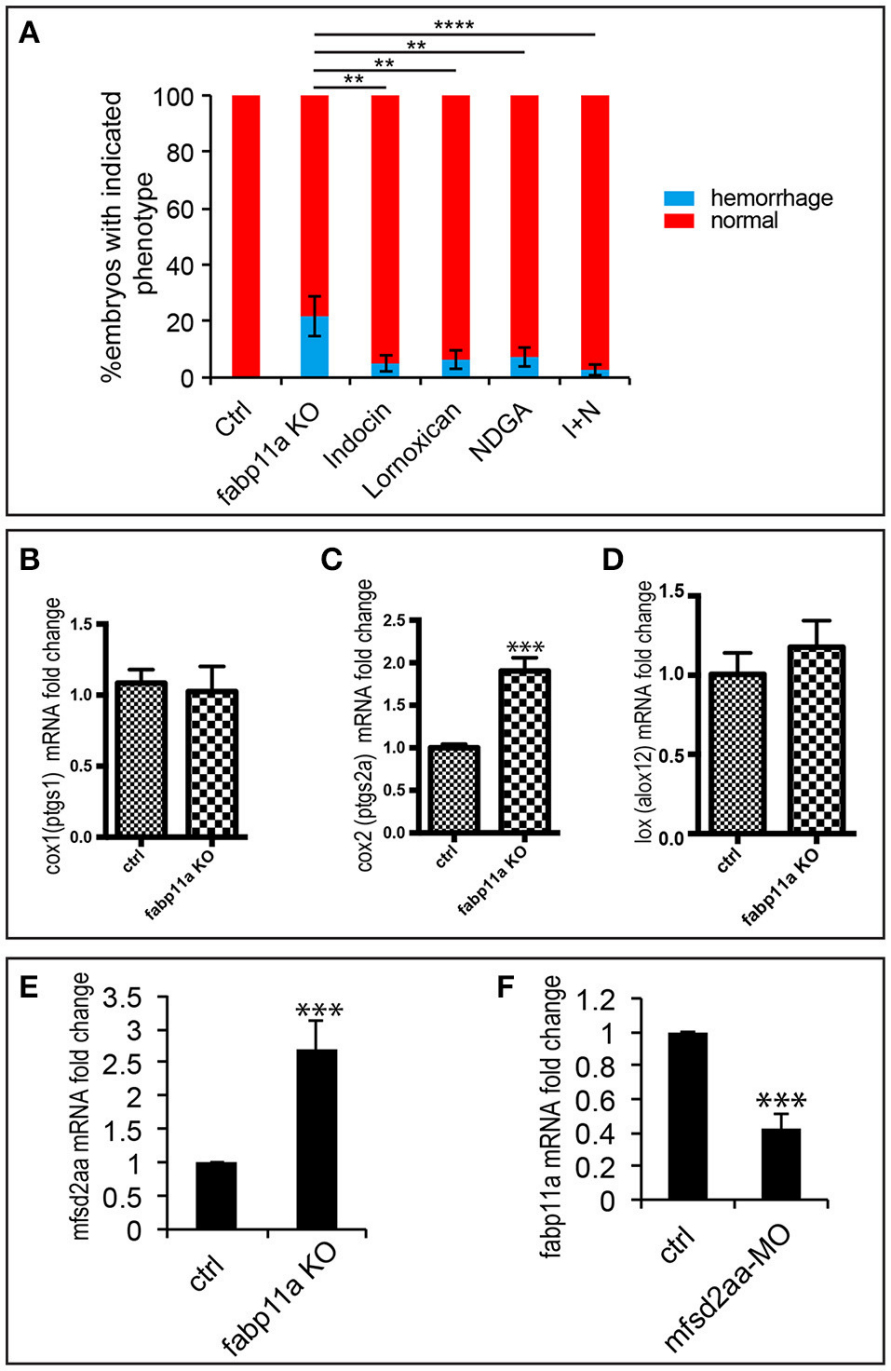
**COX and LOX inhibition partially rescued the brain vessel integrity defects caused by *fabp11a* loss-of-function. (A)** Percentage of embryos with hemorrhage phenotype in each group: control, *fabp11a* knockout, Indocin treated, Lornoxican treated, nordihyroguaria-retic acid (NDGA) treated, and Indocin and NDGA jointly (I+N) treated larvae. Fisher's exact test, ^**^P < 0.01, ^****^P < 0.0001. **(B–D)** Real-time PCR analysis of expression of *cox1, cox2*, and *lox* in control and *fabp11a* knockout embryos. *T*-test, ^***^P < 0.001. **(E)** Real-time PCR analysis of *mfsd2aa* expression in control and *fabp11a* knockout embryos *T*-test, ^***^P < 0.001. **(F)** Real-time PCR analysis of *fabp11a* expression in control embryos and *mfsd2aa* morphants. *T*-test, ^***^P < 0.001.

The role of fatty acids (FA) on blood-brain barrier (BBB) permeability is well documented. It has been reported that in rat cerebral microvessels, inhibition of COX by indomethacin and LOX by nordihyroguaria-retic acid could effectively block the endothelial barrier defects caused by arachidonic acid (AA) (Easton and Fraser, 1998). AA is a precursor for the formation of various bioactive molecules including prostaglandins, such as PGE2, and leukotrienes. Since FABPs are capable of binding a variety of hydrophobic ligands, such as long-chain fatty acids, eicosanoids, leukotrienes and prostaglandins (Haunerland and Spener, 2004; Makowski and Hotamisligil, 2005), we examined whether COX inhibition by indomethacin and Lornoxican treatment and LOX inhibition by nordihyroguaria-retic acid treatment could rescue the brain vessel integrity defects caused by *fabp11a* deficiency. As a result, we found that treatment of indomethacin/Lornoxican, nordihyroguaria-retic acid separately, and their combination significantly reduced the ratio of brain vessel integrity defects caused by *fabp11a* deficiency (Figure 5A). Additionally, we discovered that the expression of *cox2* was significantly upregulated (Figure 5C); meanwhile *lox* was slightly upregulated (Figure 5D), in *fabp11a* knockout embryos. In contrast, the expression of *cox1* was not significantly affected (Figure 5B).

During the process of lysophosphatidylcholine (LPC) synthesis, Mfsd2a could transport DHA and other needed fatty acids (Ben-Zvi et al., 2014; Nguyen et al., 2014). Given *mfsd2aa* zebrafish morphants present disruption of the blood vessel integrity (Guemez-Gamboa et al., 2015), we reasoned that *mfsd2a* is involved in the effects of FABP4 on vascular permeability. We did the expression analysis of *mfsd2aa* in the *fabp11a* mutants and *fabp11a* in *mfsd2aa* morphants by Real-time PCR, and found that *mfsd2aa* was upregulated in the *fabp11a* mutants (Figure 5E). In contrast, *fabp11a* was downregulated in *mfsd2aa* morphants (Figure 5F). However, either knockdown or upregulation of *mfsd2aa* in the *fabp11a* mutants failed to rescue the blood vessel integrity defects (Figure S3).

## Discussion

*Fabp11a* is highly expressed in the developing brain vessels of zebrafish. Currently, we revealed that *fabp11a* loss-of-function caused hemorrhage in the head and impaired brain vessel integrity for the first time.

Recently, accumulating data indicates that FABP4 was involved in the regulation of angiogenesis. Elmasri et al. reported that FABP4, which is expressed in the microvascular ECs in several normal tissues and organs, functions as a target of the VEGF/VEGFR2 signaling pathway and is a positive regulator during the mice and human endothelial cell (EC) proliferation (Elmasri et al., 2009). Consistently, It was also demonstrated that deficiency of FABP4 in human umbilical vein endothelial cells (HUVECs) and aortic rings from *FABP4*^−/−^ mice leads to attenuation of angiogenic responses, such as endothelial cell migration, proliferation, apoptosis, and morphogenesis through modulation of several pathways, including p38, eNOS and SCF/c-kit signaling (Elmasri et al., 2009, 2012; Ghelfi et al., 2013). It was also found that EC-FABP4 is strongly regulated by the mTORC1 pathway and its inhibitor rapamycin in HUVECs (Elmasri et al., 2012). Later on, extended studies from the same lab showed that FABP4 is required for the retinal development but not for normal vascular development (Saint-Geniez et al., 2014). In addition, Harjes and his colleagues showed that DLL4-NOTCH could bind NICD to FABP4 promoter regions to regulate *FABP4* gene expression directly, and endothelial NOTCH pathway is necessary for the FABP4 response to VEGFA signaling (Harjes et al., 2014). Currently we showed that chemical inhibition of p38 MAPK, eNOS and mTORC1 signaling did not cause hemorrhage in zebrafish. These results suggest that those are not the pathways responsible for brain vessel integrity defects caused by *fabp11a* deficiency. We also showed that COX and LOX inhibition partially rescued the brain vessel integrity defect caused by *fabp11a* loss-of-function, suggesting the integrity defect was relevant to the fatty acid function. We have shown that *mfsd2aa* was upregulated in the *fabp11a* mutants. However, neither knockdown nor upregulation of *mfsd2aa* in the *fabp11a* mutants could rescue the blood vessel integrity defects. These data suggests that *mfsd2aa* and *fabp11a* regulate vascular integrity in a different pathway. The upregulation of *mfsd2aa* in response to the *fabp11a* deficiency might be a compensational effect of brain vessel integrity defects.

In our previous study, we have shown that mutation of *fabp11* causes dramatic defects in zebrafish eye development (Qi et al., 2016). However, the hemorrhagic phenotypes describe in this study were found in both the *fabp11a* mutants with or without eye defects. This suggests independent manners of Fabp11a mutation in affecting hemorrhagic phenotypes and the eye defects. It was demonstrated that *fabp11a* was highly enriched in zebrafish developing brain vessel. However, *FABP4* mRNA is not detectable in adult mouse brain and brain vessels (Elmasri et al., 2009). This might be due to the differences of the developmental stages when the expression level of *FABP4* was investigated. To date, there is no evidence to show that *FABP4* knockout leads to hemorrhage in the head and brain vessel integrity defects in mice. A possible explanation is that this phenotype in mice is non-lethal and not examined so far. If this is not the case, in mice another paralog of FABP4 may play the role, as *fabp11a* of zebrafish, in regulation of vascular permeability.

## Author contributions

DL and JinZ designed the experiments, and wrote the manuscript. JieZ, JQ, SW, LP, YS, JY, ZY, YG, CW, JG, and HZ did the experiments.

### Conflict of interest statement

The authors declare that the research was conducted in the absence of any commercial or financial relationships that could be construed as a potential conflict of interest.
