# Supplementary material for: Fatty Acid Binding Protein 11a Is Required for Brain Vessel Integrity in Zebrafish
Source: Front Physiol. 2017 Apr 11;8:214. doi: 10.3389/fphys.2017.00214 (PMC5387095; doi:10.3389/fphys.2017.00214)
Supplement: Supplementary file 1 [file DataSheet1.doc]

**Supplementary data**

**Title:** Fatty acid binding protein 11a is required for brain vessel integrity in zebrafish

**Running title:** Fabp11a regulates vessel integrity

Jie Zhang^1, *^, Jialing Qi^1, *^, Shuilong Wu^2^, Lijiao Peng^2^, Yunwei Shi^3^, Jinxian Yang^4^, Zhenhua Yin^1^, Yu Gao^2^, Chengniu Wang^1^, Jie Gong^5^, Haijun Zhang^6^*,* Jingjing Zhang^2, #^, Dong Liu^3, #^

1 Medical School of Nantong University, Nantong, China

2 Affiliated Hospital of Guangdong Medical University, Zhanjiang, 524001 China

3 Co-innovation Center of Neuroregeneration, Key Laboratory of Neuroregeneration of Jiangsu and Ministry of Education, Nantong University, Nantong, China

4 Xinglin College of Nantong University, Nantong, China

5 School of life science, Nantong University, Nantong, China

6 Laboratory Animal Center, Nantong University, Nantong, China

**
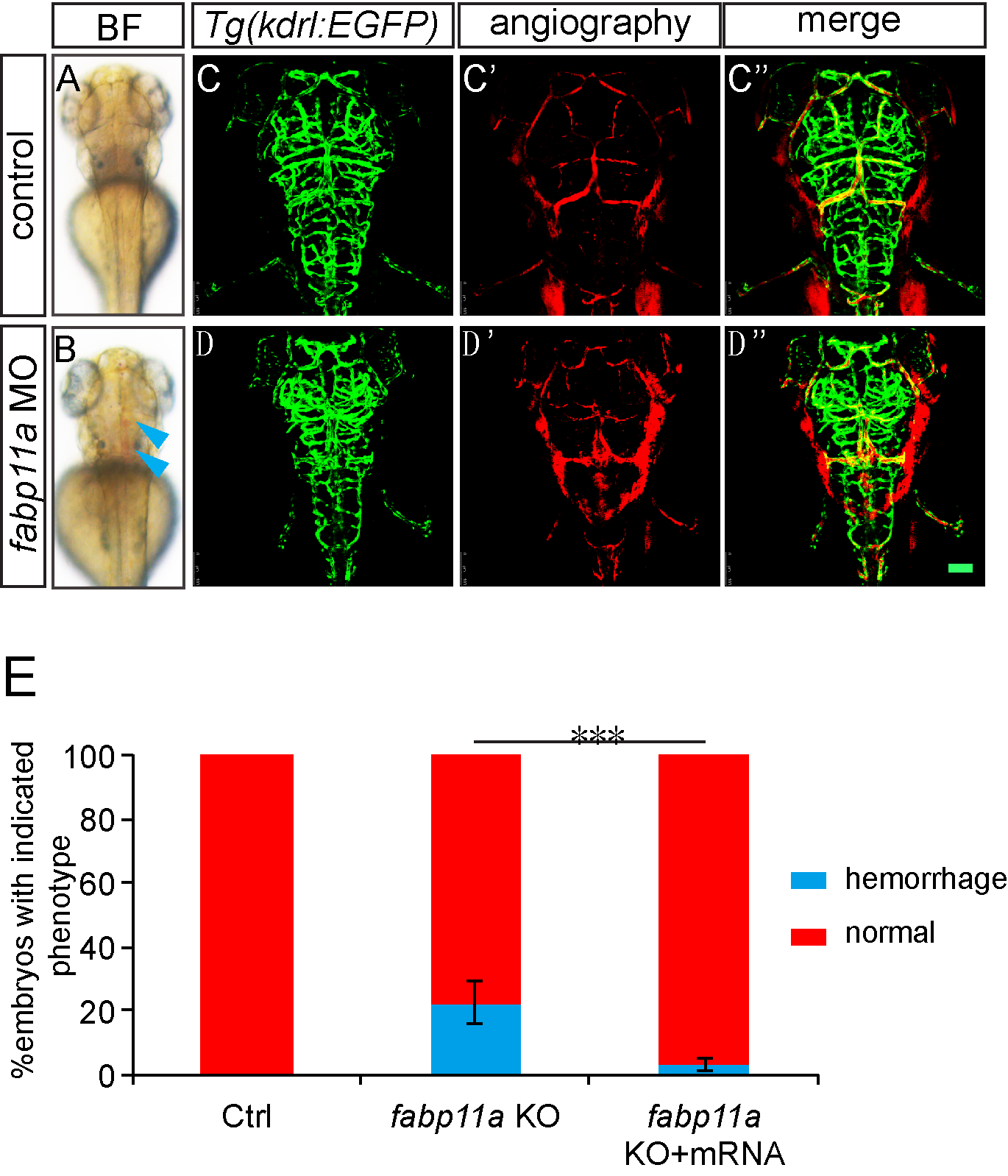
**

**Figure S1. *fabp11a* knockdown leads to zebrafish brain hemorrhage.**

(A, B) Microscopy analysis of control embryos and *fabp11a* morphants in bright field at 72 hpf. Blue arrowheads indicate hemorrhage in zebrafish head. (C-D’’) Confocal imaging analysis of *fabp11a* morphants and control *Tg(kdrl:EGFP)* embryos intracardiac injected with Rhodamine-conjugated dextran at 72 hpf. (E) *Fabp11a* mRNA injection rescued the blood vessel integrity defects caused by *fabp11a* deficiency, χ2 test, ****P*<0.001. Scale bar: 20 μm.

**
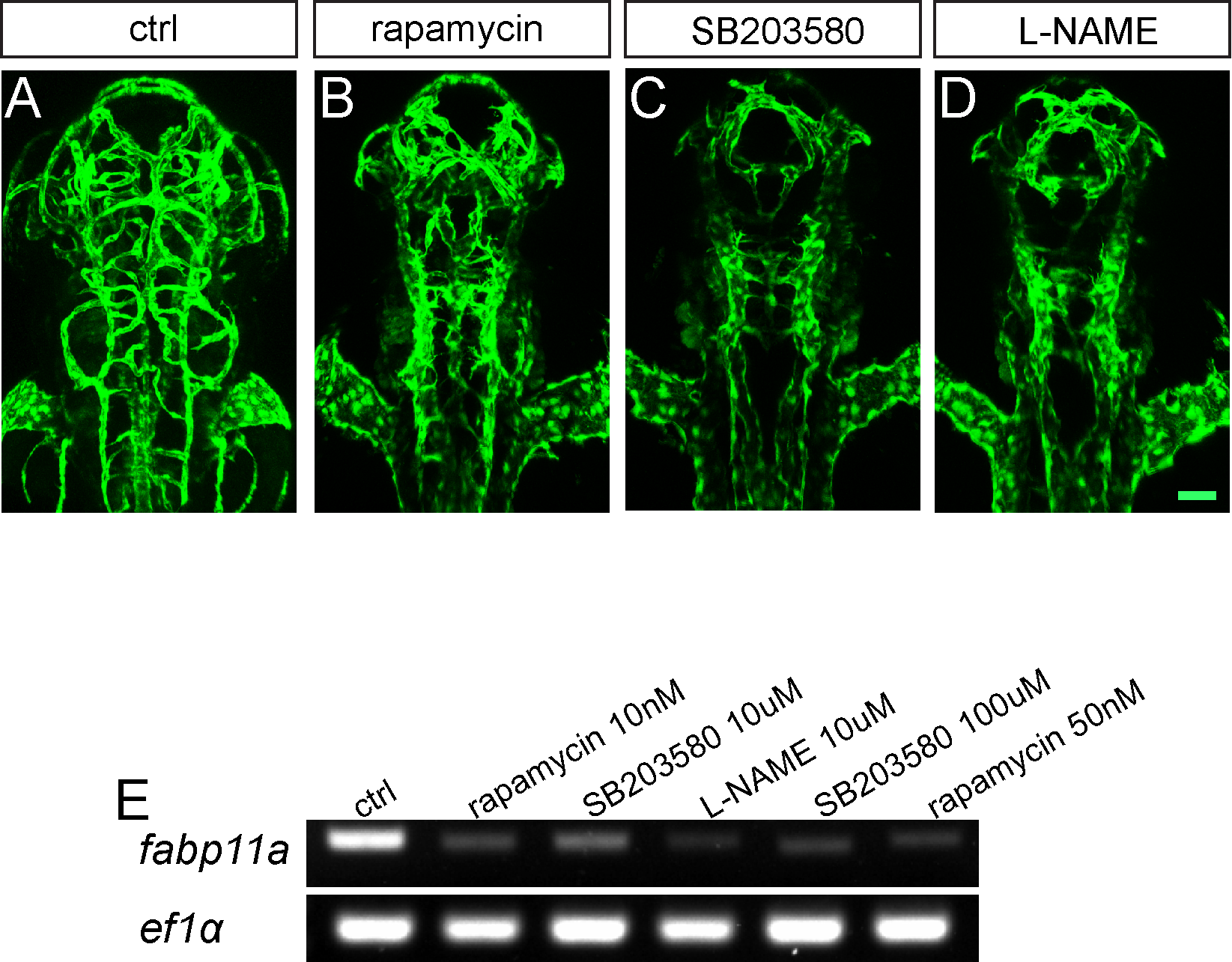
**

**Figure S2. Pharmacological inhibition of mTOR, eNOS and P38 signaling impairs branching angiogenesis of brain vessels.**

(A-D) Confocal imaging analysis of control, rapamycin treated, L-NAME treated and SB203580 treated *Tg*(*kdrl:EGFP*) embryos. (B) Reverse transcription polymerase chain reaction analysis of *fabp11a* expression in control, rapamycin treated, L-NAME treated and SB203580 treated *Tg*(*kdrl:EGFP*) embryos. Scale bar: 20 μm.


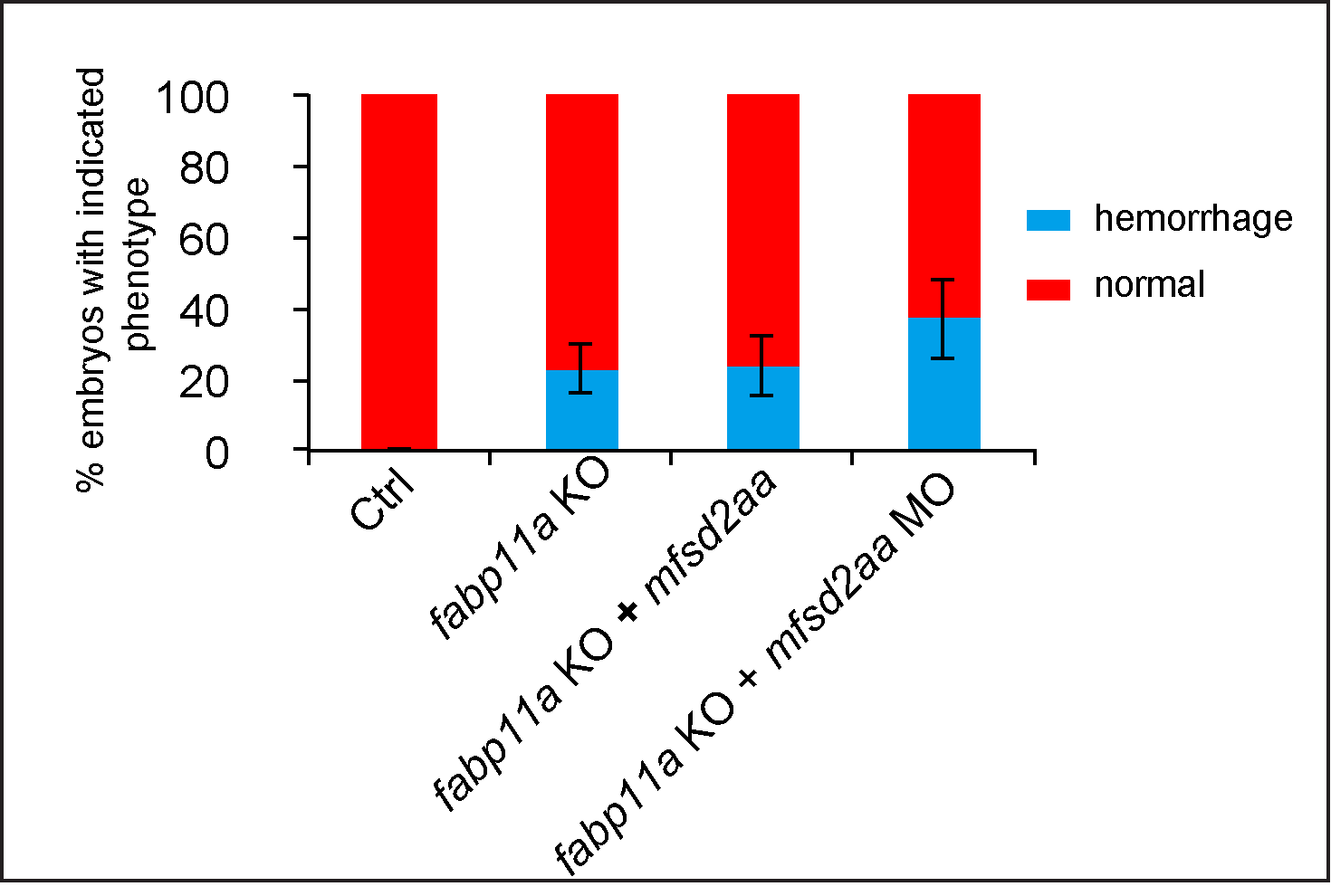


**Figure S3. Either knockdown or upregulation of *mfsd2aa* in the *fabp11a* mutants failed to rescue the blood vessel integrity defects.**

| **Supplementary Table 1. PCR primers** | | |
| --- | --- | --- |
|  | Left primer | Right primer |
| Lox1(*alox12*) | 5’- ATGATTATGGACACGCTGCC -3’ | 5’- AATCACCTTCAAAGCAGCGG -3’ |
| Cox1(*ptgs1*) | 5’- AACCCTACACATCCTTCGCA -3’ | 5’- AGCTGGGTAGAACTCCATAGC-3’ |
| Cox2(*ptgs2a*) | 5’- CAAACCAGACAGATGCGCTA -3’ | 5’- TGACCGTACAGCTCCTTCAG -3’ |
| *mfsd2aa* | 5’- TGCTTCCAGATGTGGTTGAC -3’ | 5’- CCATCCTTCATTGGTCTGGT -3’ |

**Supplementary Table 2. The Alignment Results of Zebrafish Fabp11a With That of Other Vertebrate FABP4 Protein Sequences**

| Species | Identities | Positives |
| --- | --- | --- |
| dre-xla  dre-gga  dre-mmu  dre-rno  dre-hsa | 62/134(46%)  70/130(54%)  67/130(52%)  67/130(52%)  67/130(52%) | 87/134(64%)  98/130(75%)  93/130(71%)  90/130(69%)  92/130(70%) |
